# Life Expectancy of Transformer Paper Insulation Retrofilled with Natural Ester in the Laboratory

**DOI:** 10.3390/polym15224345

**Published:** 2023-11-07

**Authors:** Andrés Montero, Belén García, Carlos López

**Affiliations:** Electrical Engineering Department, Universidad Carlos III de Madrid, 28911 Leganés, Spain; bgarciad@ing.uc3m.es (B.G.); 100382988@alumnos.uc3m.es (C.L.)

**Keywords:** transformer, oil–paper insulation, paper retrofilling, thermal ageing, natural ester, Kraft paper, paper life expectancy

## Abstract

The use of alternative insulating liquids instead of mineral oil in transformers is spreading around the world due to their superior fire resistance. Furthermore, researchers have demonstrated that these oils increase the lifespan of the solid insulation of the transformers and, thus, the life expectancy of the equipment. Retrofilling of transformers with natural and synthetic esters allows companies to benefit from the properties of using these liquids without making an investment into new machinery. This paper investigated the ageing process of Kraft paper that was retrofilled with a natural ester in the laboratory. The Kraft paper samples were subjected to accelerated thermal ageing in an oven at 130 °C, and markers such as the degree of polymerisation and tensile strength were measured. The ageing tests comprised a first period, where the samples were immersed in mineral oil, followed by a replacement of the oil with a natural ester. As moisture is a determinant factor for paper ageing, two sets of samples with different moisture contents were tested. The results showed that the retrofilling of the transformers may slow down the degradation rate of the solid insulation despite the presence of remaining mineral oil adsorbed in the paper.

## 1. Introduction

Retrofilling transformers with natural and synthetic esters has emerged as a promising solution to enhance the safety of the electric system. Esters possess favourable electrical and fire properties. These attributes make esters a good choice to improve the operational safety of transformers. Moreover, retrofilling transformers with alternative oils reduces insurance costs, limits fire risk, and may extend the life expectancy of transformers [1,2]. It has been proven in the laboratory [3,4,5,6] that the presence of esters slows down the ageing rate of cellulose due to the higher hygroscopicity of these liquids and the hindrance of glucose chain cleavage by transesterification reactions. As a result of the reduced ageing rate of cellulose in the presence of esters, transformer insulation can safely withstand higher temperatures. This is important since the viscosity of esters is higher than that of mineral oils and, thus, the temperatures reached in the solid insulation may be higher if these materials are used as a liquid insulation.

Although there is agreement on the fact that cellulose insulation ages more slowly in the presence of an ester, it is not so clear that this result can be extrapolated to the insulation of retrofilled transformers. Experimental evidence on this fact is mainly based on laboratory accelerated ageing tests carried out on cellulose–ester systems, but the liquid insulation of retrofilled transformers is a mixture of mineral oil (MO) and natural (NE) or synthetic ester [7,8,9], the MO being the material that remains mostly adsorbed in the paper [10,11].

McShane et al. [3] conducted a study to investigate the ageing process of thermally upgraded Kraft (TUK) paper samples subjected to retrofilling with NE in the laboratory. The experiments involved ageing tests on TUK paper exposed to MO, NE, and retrofilling. The samples were dried to an initial moisture content of 0.5% and aged at 160 °C and 170 °C for 3000 h. Periodic measurements were taken of the moisture content (MC), degree of polymerisation (*DP*), tensile strength (*TS*), and furanic compound concentration. The results showed that the retrofilled samples displayed a change in the ageing trend, resembling the ageing rate of NE-exposed TUK paper in terms of the *DP* and *TS*, while both NE and retrofilled samples exhibited significantly lower furanic compound content compared to MO-exposed samples. Although the conclusions of the paper are relevant, the experimental conditions included in the study were limited, and some aspects need further investigation, such as the role of moisture in the ageing process. Additionally, the ageing temperatures considered by the authors were above the temperatures that may be reached in the insulation of in-service transformers, which can change the chemical reactions involved in the ageing process.

This paper carried out an experimental study to further evaluate the ageing process of cellulose insulation in NE-retrofilled Kraft paper. The experiment compared the ageing rate of the Kraft paper in the presence of MO and NE and under a retrofilling scenario at a temperature of 130 °C, while carrying out an exhaustive control of the physical–chemical markers of the solid and liquid materials during the tests. As moisture is one of the agents with a greater impact on the ageing processes of cellulose and is also an element that behaves in a different manner in MO- and NE-based insulation, two moisture contents of the paper samples were considered.

## 2. Objective and Scope of the Ageing Study

The experimental ageing study was designed to investigate the influence of the retrofilling process on the ageing rate of the transformer’s solid insulation. To achieve the objective, the study included accelerated ageing tests on three types of oil–paper insulation systems:

1. Kraft paper immersed in MO.
2. Kraft paper immersed in NE.
3. Kraft paper aged for some time in the presence of MO and, then, after the replacement of the MO with NE, aged in the presence of the NE.

The first and second systems emulated the ageing process of the MO- and NE-impregnated Kraft paper, whereas the third reproduced the ageing process of the insulation of a retrofilled unit (i.e., the paper had experienced some ageing in the presence of the MO, and then, the insulating liquid was replaced by an NE, although the solid insulation remained impregnated in the MO).

To analyse the impact of moisture in the ageing process, the study was repeated considering two different moisture contents for the solid insulation.

## 3. Materials and Methods

The ageing tests were carried out on samples of Kraft paper and insulating liquid fit in crystal vials. The materials in the vials were subjected to thermal ageing in an oven for different times, aiming at monitoring the progress of the ageing process. This section details the materials and methods used to prepare the samples for ageing, the development of the ageing tests, and the physical–chemical evaluation carried out on the samples before and after the ageing process.

### 3.1. Preparation of the Samples

**Figure 1:**
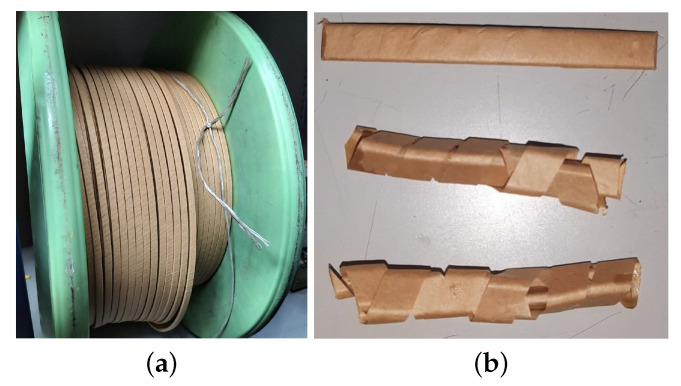
Paper samples used for the ageing tests. (**a**) Coil of insulated flat copper wire. (**b**) Paper-insulated flat copper wire (**upper**) and paper specimens (**middle** and **bottom**).

The solid insulation considered for the ageing tests was Kraft paper, which is used to wrap the copper wire, which constitutes the transformer windings. This type of thin insulation is the one subjected to higher thermal stress in the transformer and is also one of the most-critical elements for the safe operation of the equipment. The paper was extracted from a coil of insulated flat copper wire (Figure 1a) supplied by the manufacturer Vicente Torns (Rubí, Spain). The paper had a thermal conductivity 0.16 W/m°C and a thermal index 105 °C.

Strips of Kraft paper with a total mass of 2.544 g were placed in each ageing vial. Additionally, aiming at considering the effect of copper in the ageing process, a paper-insulated copper wire of a mass of 9.5 g was also fit in each of them. Figure 1b shows the taped copper plates and Kraft paper strips used for the accelerated ageing tests. The vials were then filled with 100 mL of oil for impregnation. The total mass of oil in each vial was 89.5 g.

Prior to impregnating the Kraft paper, it was subjected to drying. Samples with two different initial moisture contents were prepared in order to assess the impact of water on the degradation rate of the paper. It has been proven that the water content of the paper has a major impact on the degradation reactions of cellulosic materials [5,12].

Some of the vials were subjected to drying in a vacuum oven for 24 h at 70 °C and 1 mbar, followed by an additional drying period of 2 h at 90 °C and 1 mbar. Subsequently, half of the vials were impregnated with MO or NE, forming the so-called high-moisture (HM) group, whose initial moisture content was approximately 4.5% by weight for the MO-impregnated samples (such high moisture contents are not commonly found in modern transformers). The remaining vials were kept in the vacuum oven at 70 °C and 1 mbar for 96 h, followed by another 2 h at 90 °C and 1 mbar. After this period, they were impregnated with MO or NE, resulting in an initial moisture content of around 1% in weight for the MO-impregnated samples, forming the so-called low-moisture (LM) group. The initial moisture for the NE-immersed samples was a little higher due to the hygroscopic character of the NE.

After the drying process, the vials were filled with 100 mL of NE or MO. The MO used for the tests was Nytro 4000X (Nynas, Stockholm, Sweden), and the NE was FR3 (Cargill, Wayzata, MN, USA). The oils were also dried under vacuum at 60 °C prior to filling the vials. The initial moisture content of the liquids was 9 ppm for the MO and 154 ppm for the NE. Finally, the ageing vials were sealed with a silicone septum and treated with bubbling nitrogen for one minute. The nitrogen treatment was used to limit the content of oxygen in the vials and avoid excessive oxidation of the materials during the tests.

The vials filled with the NE were employed to analyse the ageing process in the presence of this substance. As for the vials containing the MO, a portion of them were utilised for investigating the ageing process of the Kraft paper in this oil, while the others, following an initial ageing period, underwent an oil change from MO to NE, allowing us to examine the ageing process of the solid insulation in the retrofilled paper.

**Figure 2:**
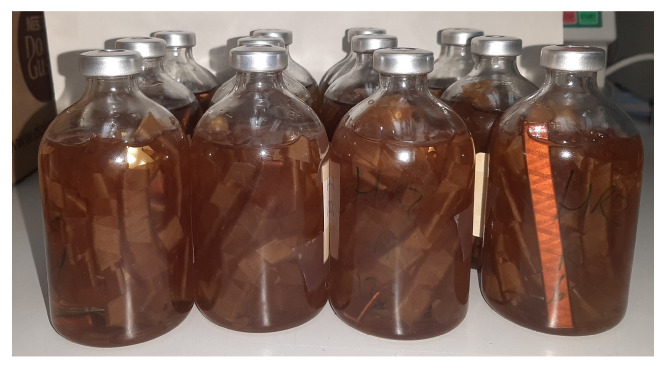
Ageing vials.

The samples that were subjected to retrofilling were sealed with a new septum after changing the oil and subjected to further nitrogen bubbling before putting them back into the ageing oven. Figure 2 shows some of the testing vials filled with oil and sealed with a septum after completing the preparation process.

### 3.2. Ageing Process

The ageing tests were carried out in an oven at a temperature of 130 °C. The chosen temperature was within the temperature range defined by IEC 60076-7 [13] for long-term overloads (i.e., 140 °C); thus, the chemical reactions involved in the ageing tests were similar to those that appear in overloaded transformers. The testing times were determined taking into account previous experiences of the authors with accelerated ageing in the presence of MO and NE [5] and based on the results reported in the literature [3]. All the samples were subjected to a first ageing period after which the retrofilling was carried out on a set of MO-filled samples. That initial period lasted 218 h for samples of the HM group and 386 h for the samples of the LM group.

For retrofilling the vials, the MO was first drained, followed by rinsing the paper with a small amount of NE, which was then discarded. Finally, it was refilled with dry NE to simulate the ageing process of the insulation system of a transformer with moderately aged solid insulation subjected to retrofilling.

**Figure 3:**
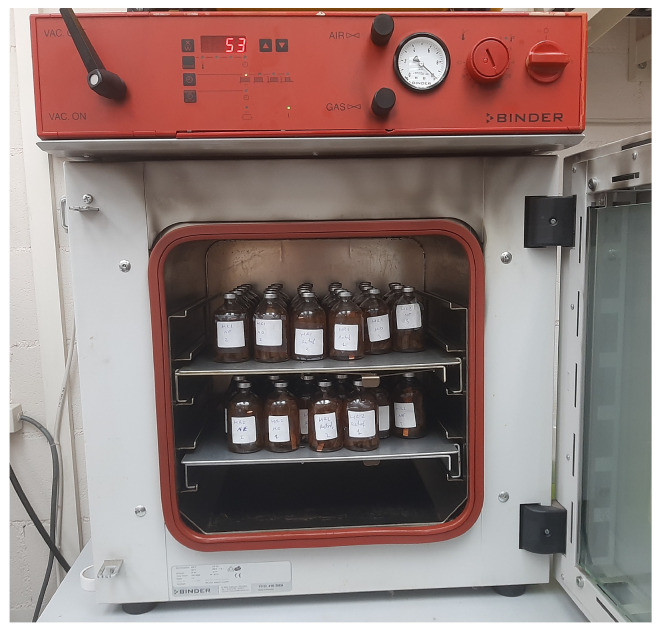
Vials inside the ageing oven.

After the retrofilling, the three batches of samples remained at 130 °C until the end of the ageing tests, which lasted 2400 h. Figure 3 shows the vials inside the ageing oven.

The vials were periodically extracted from the oven to obtain samples with different degrees of ageing and to observe the evolution of the ageing markers. Table 1 shows the extraction times for the vials of the different kinds of samples with high and low moisture contents. These times will be used to represent the evolution of the different markers in Section 4. The retrofilled samples in the HM group were firstly aged in the presence of MO for 218 h, whereas this time increased up to 386 h in the set of LM samples.

### 3.3. Sample Testing

The Kraft paper samples and oil in the vials were subjected to laboratory testing to evaluate the evolution of the different parameters for different ageing times.

To characterise the ageing condition of the paper, the *TS* and *DP* were measured. The DP characterises the average length of the glucose chains, while the *TS* determines the mechanical properties of the paper. Both properties are related to the ageing degree of cellulose. It is known that thermal ageing reduces the length of the glucose chains that compose the paper and that the chain shortening makes the paper more brittle (i.e., reduces its tensile strength). The *DP* of a new paper is typically around 1200. IEEE Std C57.91-1995 [14] establishes that a paper is at its end-of-life if its *DP* reaches a value of 200 or if it retains just 25% of its initial *TS* value. The end-of-life of transformers has also been shown to occur when the paper reaches a *DP* of 100, rather than 200 [15].

The *TS* of the samples was measured with a Vertical Universal Tensile Tester MTC-100, IDM (Spain). Ten paper strips of a length of 100 mm were extracted from each ageing vial and tested with the tensile strength tester. The parameters used for the test were adjusted to ISO 1924-3:2005 [16]. The obtained *TS* data were statistically treated by discarding the outlier measures, considering a 95% confidence level, and calculating the average value of the remaining values. The *DP* was measured according to Std. IEC 60450:2004 [17] in the laboratory of the company CEIS (Spain).

Additionally, the water contents of the liquids and those of the cellulose samples were measured by the Karl Fischer (KF) method, according to IEC-60814 [18], using a KF Coulometer Methrom 831 combined with a KF Thermoprep oven.

Finally, the acidity of the liquids was also measured according to Std IEC 62021-3 [19] using the automatic potentiometric titrator 848 Titrino Plus (Metrohm, Herisau, Switzerland).

The evaluation of the paper ageing by-products was carried out by the measure of the furanic compounds and the phenol content, although the results of these tests are not presented in this paper.

## 4. Results

### 4.1. Evaluation of the Paper Degradation

**Figure 4:**
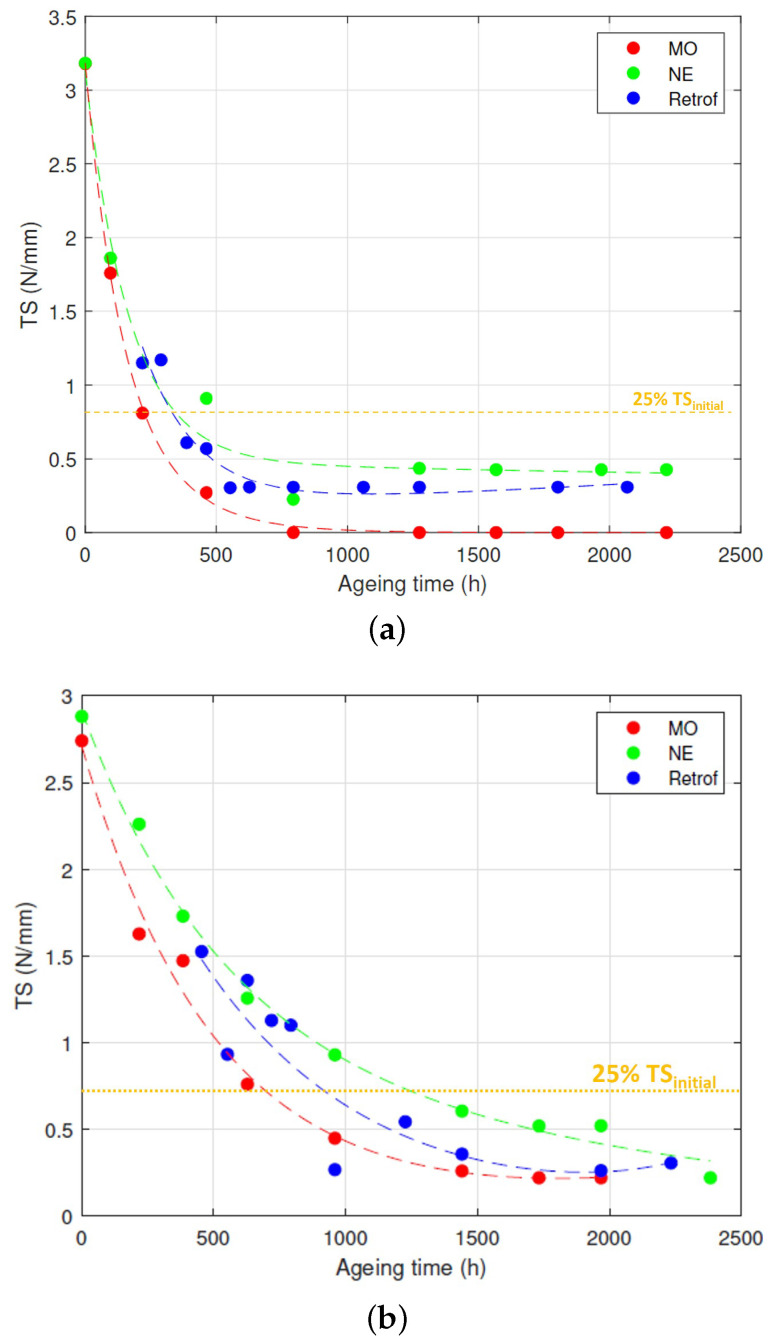
Tensile strength. (**a**) High moisture. (**b**) Low moisture.

**Figure 5:**
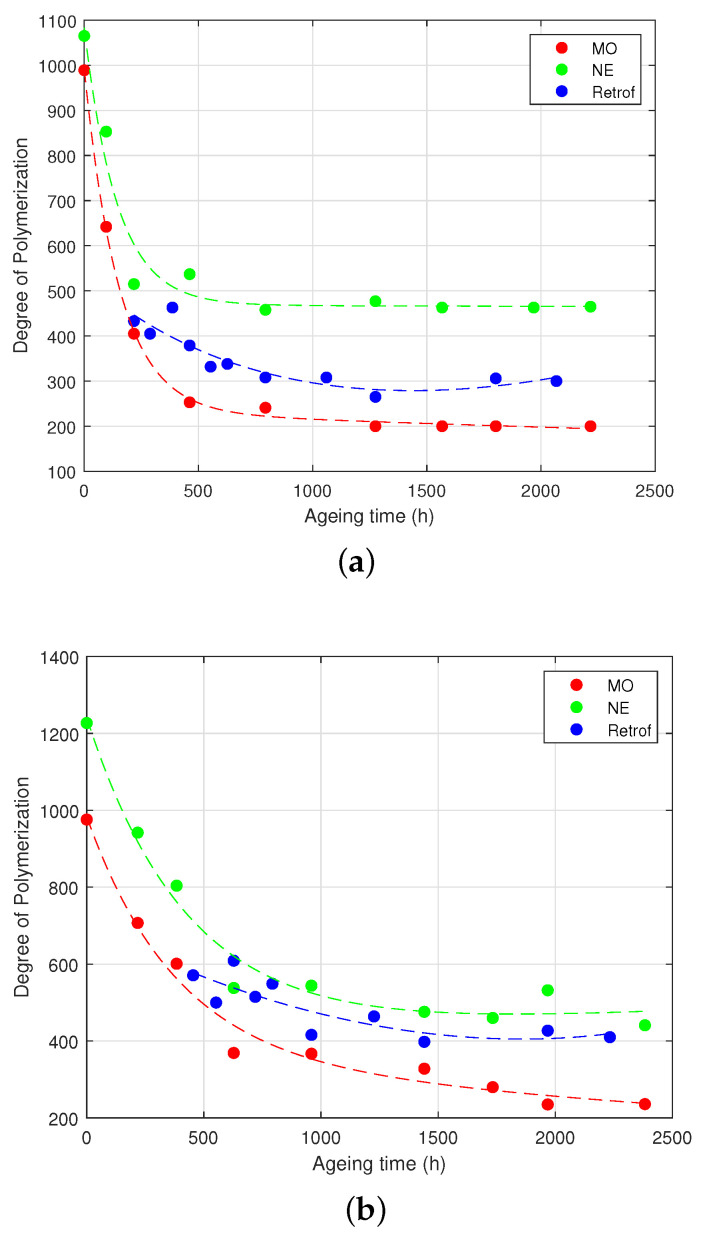
Degree of polymerisation measured at different times of the test. (**a**) High moisture. (**b**) Low moisture.

The condition of the paper at different stages of the test was analysed with the measurements of the *TS* and *DP*. Figure 4 and Figure 5 show the results of the two tests.

Before analysing the ageing results globally, it is interesting to evaluate what was the condition of the Kraft paper when the retrofilling was carried out. Table 2 shows that the traction resistance of the Kraft paper in the HM group was only 36.1% of the value measured at the beginning of the tests. On the other hand, the LM samples retained 71.1% of the *TS* at the time of carrying out the retrofilling. The estimations of the remaining life according to the *DP* were similar: 43.8% for the HM samples and 71.8% for the LM. The results evidenced the huge influence of the moisture content on the ageing rate of the paper.

Figure 4a shows the evolution of the *TS* of the paper over time for the HM group. A quick degradation was observed at the first 500 h of the tests for both the MO-impregnated Kraft paper and the NE-impregnated one. The *TS* reached 25% of its initial value after 210 h of ageing for the MO-immersed paper and 390 h for the NE-immersed samples. In fact, some of the MO samples could not be measured after 800 h because it was not possible to extract the strips from the vial without breaking them. The retrofilling reduced the degradation rate in comparison to the MO-immersed samples. As can be seen, those samples followed a similar ageing rate as the NE-immersed paper Kraft paper, reaching 25% of the initial *TS* after 380 h.

Figure 4b shows the variation of the *TS* over the ageing time for the LM group. As the initial moisture in the paper was lower than in the previous case, the degradation rate was lower in the three cases. The MO, NE, and retrofilling cases reached 25% of their initial *TS* at the times of 710, 1237, and 935, respectively.

The results of the three ageing scenarios for each moisture content were fit to the sum of two exponential functions according to (1).

(1) $$TS=a\cdot e^{\left(b\cdot t\right)}+c\cdot e^{\left(d\cdot t\right)}$$

where *TS* is the tensile strength, *t* is the ageing time, and *a*, *b*, *c*, and *d* are the fitting coefficients.

The values of the parameters obtained for each case are shown in Table 3. The fitting was very good for the cases of the MO- and NE-immersed samples, with a value of $R^{2}$ close to 1. The adjustment was a little worse for the case of retrofilled samples with both high and low moisture contents.

The other parameter to measure the degradation level of the paper, the *DP*, is plotted in Figure 5 vs. the ageing time. As can be seen, the *DP* followed a similar pattern to the *TS*: the samples of the HM group (in the presence of both MO and NE) showed a much faster degradation than the samples with lower initial moisture.

Additionally, it can be seen that the retrofilled samples followed a similar degradation tendency as the NE-immersed samples for the low-moisture-content group. In the case of the HM group, the paper was very degraded when the retrofilling was performed, and thus, a smaller improvement was observed. However, it is clear that the degradation rate of those samples was also slower than that of the MO-immersed samples with similar moisture contents.

Figure 5a illustrates the evolution of the *DP* of the different samples over the ageing time for samples belonging to the HM group. In both the MO- and NE-impregnated paper, a significant initial decline in the *DP* occurred due to rapid ageing, taking place within approximately 500 h of ageing. For samples treated with the MO throughout the ageing process, a *DP* of 200 was achieved at 1800 h. In contrast, the *DP* of the NE-impregnated samples remained steady at around 470 after 665 h of ageing, without reaching the *DP* of 250, as well as the retrofilled samples, which stabilised at around a *DP* of 280 for the remaining ageing duration.

Figure 5b illustrates the progression of the *DP* for the LM group over the ageing time. It was evident that the degradation rate in the LM group was slower compared to the HM group. In the LM group, the retrofilled samples exhibited a degradation trend that was more similar to the NE-impregnated Kraft paper samples, and they did not reach the *DP* of 200. The MO-impregnated Kraft paper samples did not reach the end-of-life marker, and its degradation was quite slower with respect to the same samples in the HM group, emphasising the effect of moisture in the solid insulation. According to [15], in the laboratory results, the paper never reached a *DP* < 200.

The *DP* also followed the same tendency as the *TS* (i.e., the sum of two exponential functions):

(2) $$DP=a\cdot e^{\left(b\cdot t\right)}+c\cdot e^{\left(d\cdot t\right)}$$

The fitting coefficients to (2) are shown in Table 4. As in the case of the *TS*, the fittings were quite precise for the MO- and NE-immersed samples, but not for the retrofilled samples.

**Figure 6:**
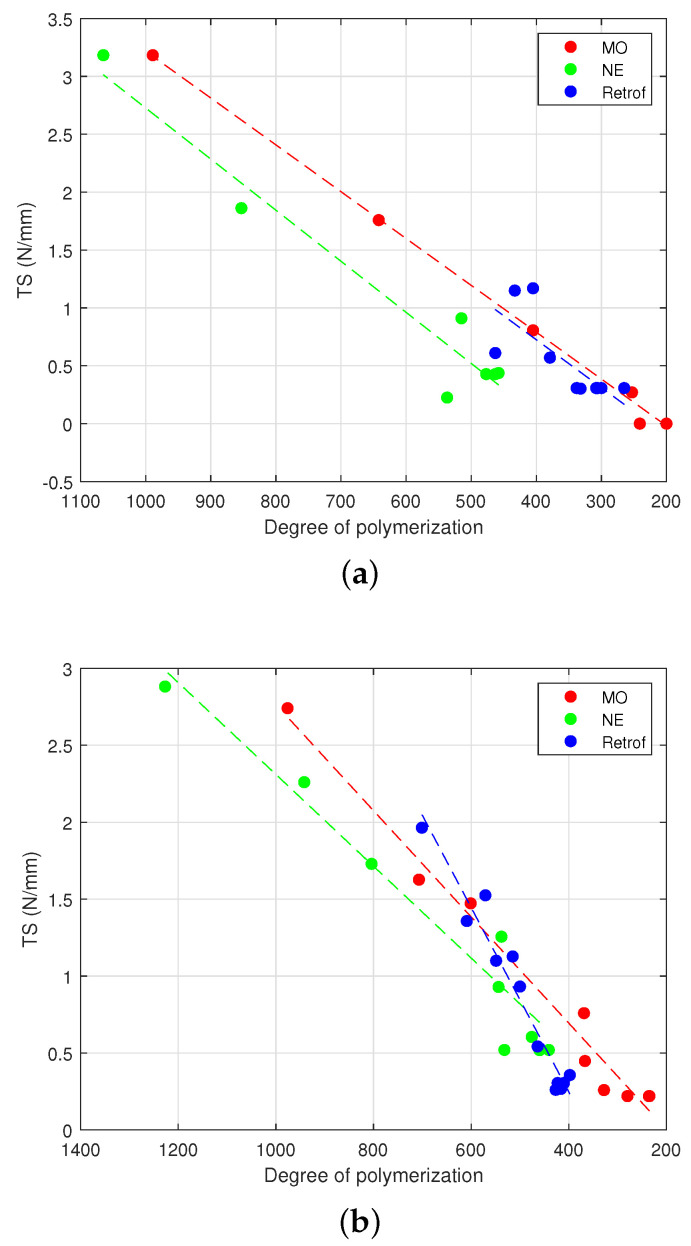
*DP* vs. *TS*. (**a**) High moisture. (**b**) Low moisture.

To complete the analysis, Figure 6 represents the evolution of the *TS* and the *DP* in the different ageing scenarios analysed in the tests. Table 5 shows the mathematical equation and Pearson’s linear coefficients ($R^{2}$); as can be seen, there was a clear correlation between both variables in all cases for each liquid. However, it must be highlighted that the limit value of the *TS* was reached at different values of the *DP* for the MO- and NE-immersed samples (i.e., 350 and 500, respectively), which suggests that the relation between the *TS* and *DP* was not the same in both materials. In the samples subjected to retrofilling, the values of the *TS* and *DP* were not well correlated.

As a general conclusion, the experimental results showed that the degradation rate of the Kraft paper subjected to retrofilling changed from that of the MO-impregnated Kraft paper to another rate that resembled more the ageing rate of the NE-impregnated Kraft paper. This result suggests that the lifespan of the solid insulation of a retrofilled transformer (and, as a consequence, the lifespan of the equipment) may be extended thanks to the replacement of the liquid insulation by an NE, despite the presence of the remaining MO adsorbed in it.

### 4.2. Evolution of the Moisture Content in Paper and Oil

**Figure 7:**
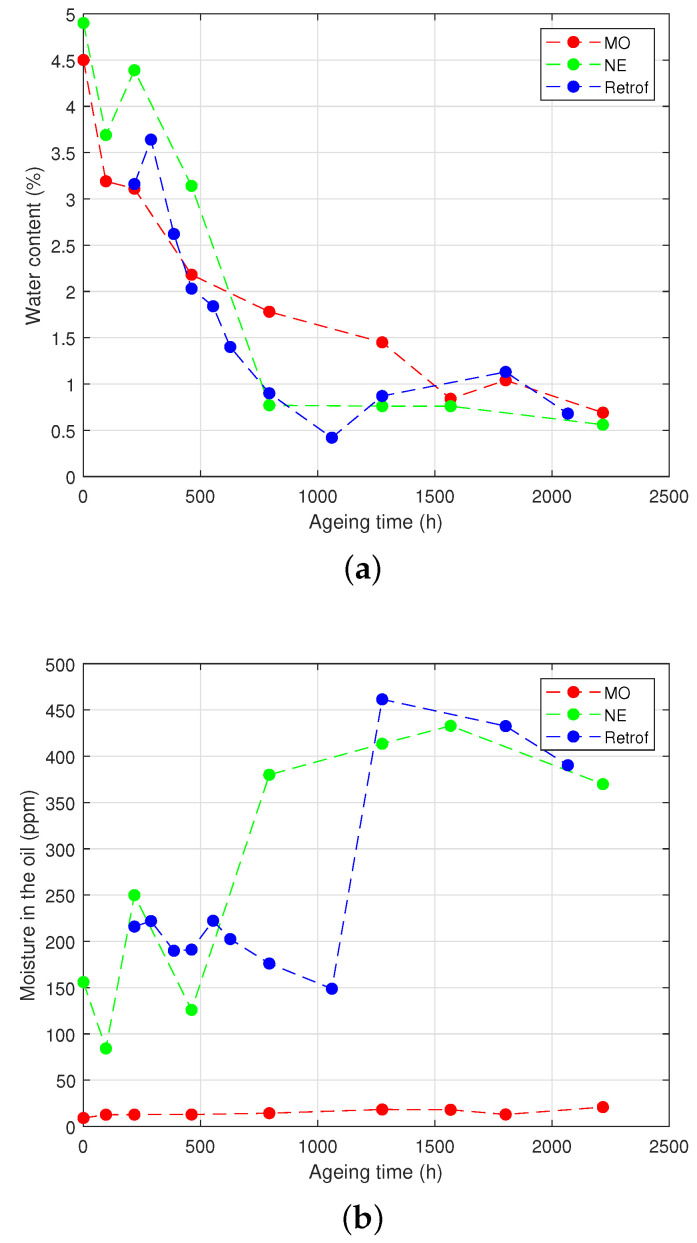
Moisture analysis of the HM group. (**a**) Moisture content in the paper. (**b**) Moisture in the oil.

**Figure 8:**
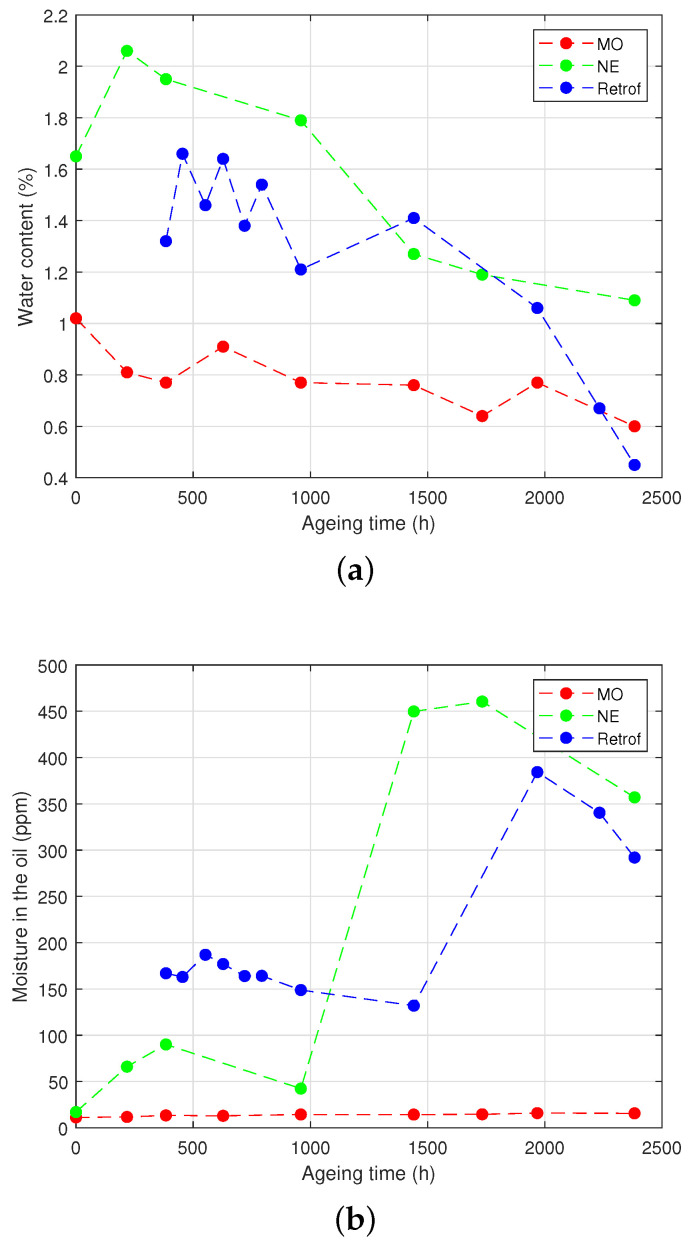
Moisture analysis of the LM group. (**a**) Moisture content in the paper. (**b**) Moisture in the oil.

The measurements of the moisture content in the Kraft paper and in the oil of the different vials are shown in Figure 7 for the HM group and in Figure 8 for the LM group.

Regarding the HM group, it can be seen that the moisture content of the paper decreased quickly during the first third of the ageing process both in the samples with the NE and the retrofilled ones (Figure 7a). This was clearly motivated by the high hygroscopic character of the NE: the water migrated from the solid insulation towards the liquid when the temperature rose until it reached the equilibrium between both media.

The case of the MO-immersed samples was more difficult to interpret. Despite that the decrease was slower than the others, the moisture content in the presence of the MO was not expected to drop so much. An explanation may be that, as the septum was punctured to inject the nitrogen in the head-space of the vials during the process of sample preparation, a small leak remained in the septum, which allowed some release of water from the vials. The amount of water in the oils (Figure 7b) tended to decrease at the beginning of the ageing for the NE-immersed and the retrofilled samples, but at certain a point, it increased drastically. An increase in the water content of the MO was also observed, although the variation was not clearly appreciated and without the step-up shown for the other samples.

The LM group (Figure 8) showed similar behaviour in the NE-filled vials. In the case of the MO-immersed samples, the water content remained more stable throughout the experiment.

**Figure 9:**
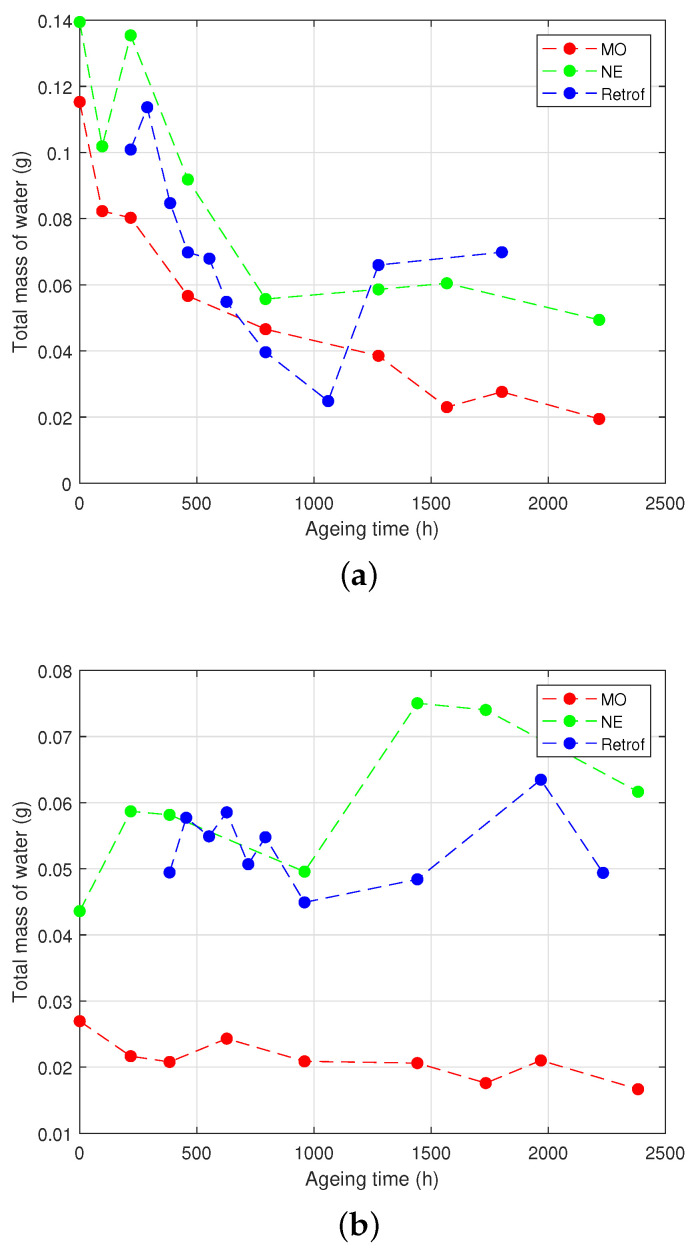
Total water content in the paper–oil system. (**a**) High moisture. (**b**) Low moisture.

Figure 9 shows the evolution of the total mass of water in the vials throughout the experiment, calculated by (3):

(3) $$mw_{t}\left(g\right)=\frac{MC_{p}\left(\%\right)\cdot m_{p}\left(g\right)}{100}+\frac{MC_{o}\left(\mathrm{ppm}\right)\cdot m_{o}\left(g\right)}{10^{6}}$$

where *mw*${}_{t}$ represents the total mass of the water, in grams, contained in a vial; *MC*${}_{p}$ and *MC*${}_{o}$ stand for the moisture content of the paper and the oil (in % in weight or ppm); and *m*${}_{p}$ and *m*${}_{o}$ are the mass of the paper and oil present in each vial, measured in grams.

As aforementioned, the differences in the initial water contents measured in the samples filled with the NE and MO, especially in the LM samples, might be due to the relatively high water content of the NE that was used to fill the vials. In the case of the HM samples, the influence was smaller, since the large mass of water of the paper was much higher than the water contents of the NE and MO.

Some mechanisms to justify the evolution of the water in the different testing vials during the experiments are proposed in Section 5. However, the migration of the water from the paper to the oil, hydrolysis, and transesterification cannot fully explain the moisture contents in ppm observed in the NE and MO.

### 4.3. Acidity and Colour of the Liquid Insulation

The acid value of the analysed samples was also measured throughout the testing time. It is important to note that the acid value of NEs is typically much higher than that of MOs. Furthermore, the acidity of NEs increases sharply when it ages, although this does not necessarily have negative implications.

**Figure 10:**
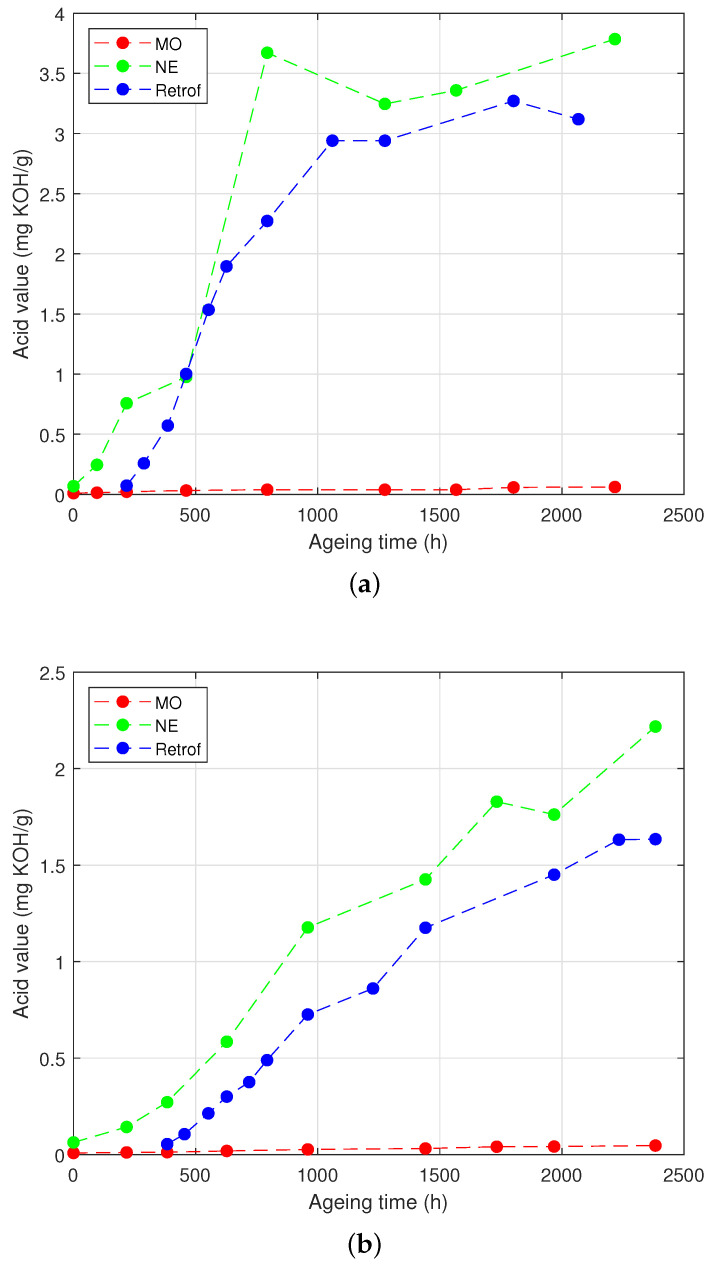
Acidity of the oil. (**a**) High moisture. (**b**) Low moisture.

Figure 10a depicts the evolution of the acid value of the different insulating liquids over the ageing period in the HM group, and Figure 10b illustrates it for the LM group. In the first set of samples, the acid value of the MO increased by 6-times, going from 0.010 to 0.061 mgKOH/g, whereas the NE samples experienced a 55-fold increase (i.e., from 0.0680 to 3.7854 mgKOH/g). Rather than focusing solely on these increments, it is important to understand the underlying processes that will be discussed in Section 5.

Figure 10b shows the variation of the acid number in the samples of the LM group. In comparison to the samples of the HM group, the values for the NE were lower in this case (the final value, 2.21 mgKOH/g, was only 35-times higher than the initial one, 0.063 mgKOH/g). In the MO samples, there was also an increase in the acid value, which was lower than in the HM group (i.e., the acidity was multiplied by five).

**Figure 11:**
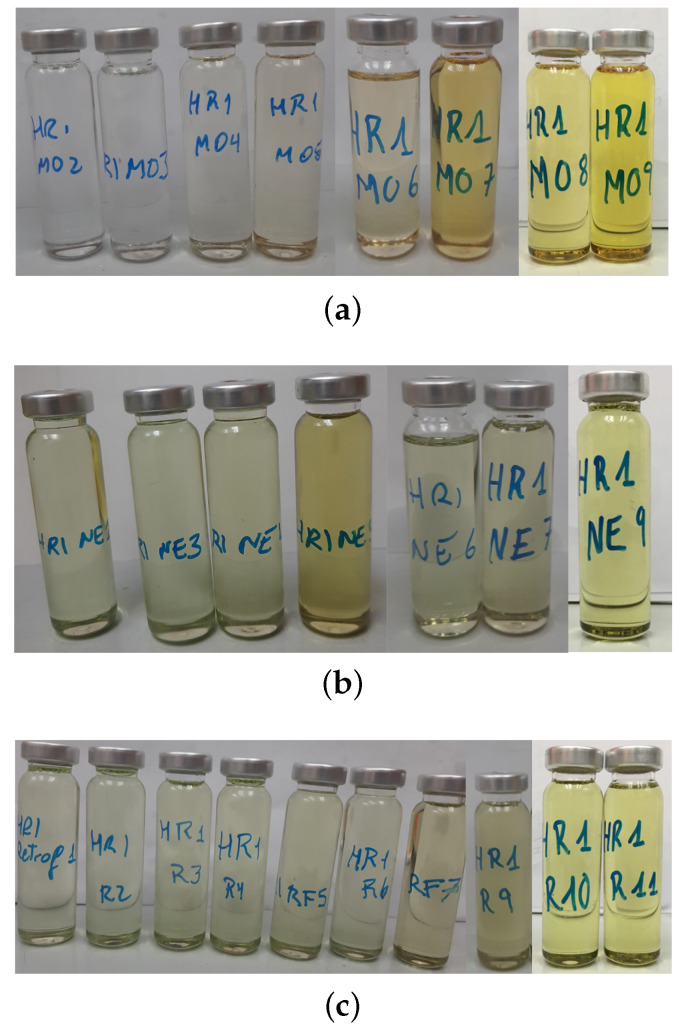
Difference in the colour of the oils in the vials during the ageing. High-moisture samples. (**a**) Mineral oil. (**b**) Natural ester. (**c**) Retrofilling.

Figure 11 shows the variations in the colour for the oil throughout the ageing process in each ageing scenario (MO, NE, and retrofilling) for the HM. The results for the LM samples were quite similar. The change of colour in the MO-immersed samples was more pronounced than in the NE-immersed samples and the retrofilled ones, transitioning from transparent to dark yellow by the end of the ageing process. The change in the colour of the NE was relatively minor, resulting in a slight darkening of its green colour.

## 5. Global Analysis of the Ageing Process in Retrofilled Samples

**Figure 12:**
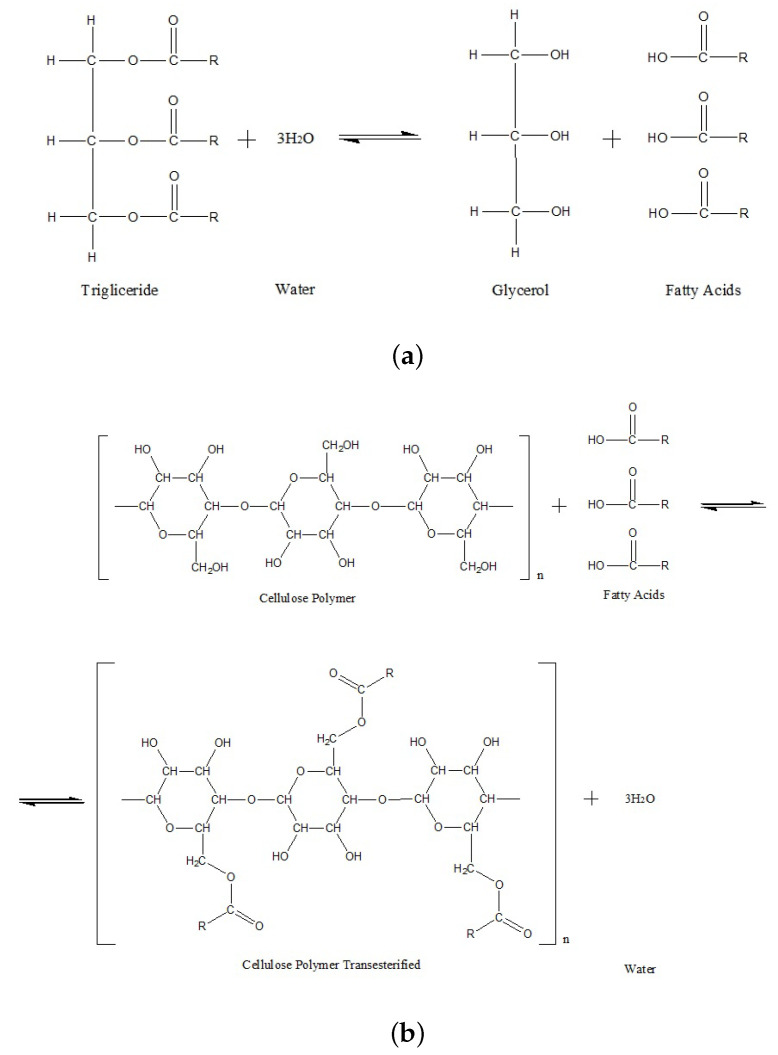
Hydrolysis and transesterification (Figures taken from [5]). (**a**) Hydrolysis reaction of esters. (**b**) Transesterification of paper chains.

A detailed description of the ageing process of the Kraft paper immersed in the NE is provided in [5]. In the presence of moisture and a high temperature, the NE undergoes a hydrolysis reaction. This chemical reaction consumes water and generates long-chain fatty acids (Figure 12a). As water is consumed, the moisture equilibrium between the solid and liquid insulation is broken, promoting additional migration of water from the solid insulation towards the paper. The presence of water will catalyse the hydrolysis of the NE, generating more fatty acids as a by-product. The fatty acids may interact with the hydroxyl groups of the cellulose molecules, adhering to the cellulose structure and generating water molecules in a reaction called transesterification (Figure 12b). Transesterification has the potential to improve the stability of paper as the presence of free fatty acids on the cellulose structure hinders the cleavage of glucose chains [20,21].

To evaluate the impact of the retrofilling on the ageing process of the solid insulation, it is important to understand whether the ageing process of the oil–paper insulation system of the retrofilled samples aligns with that observed in the NE-immersed ones or if it resembles the ageing process of the MO-immersed ones. To answer this question, it is important to analyse the results of all the measured parameters globally.

In terms of the *DP* and *TS*, a change in the trend was observed after conducting the retrofilling, transitioning from an ageing rate similar to that of the MO to a rate that resembled the one of the NE-immersed insulation (Figure 4 and Figure 5).

The effect of hydrolysis and transesterification on the water content can be seen in Figure 7, Figure 8 and Figure 9. At the beginning of the ageing, the samples with the NE did not show a clear trend. In the case of the retrofilled samples, the amount of water in the oil dropped due to the hydrolysis reactions, where water was consumed. As ageing progressed, a sharp increase of the water content in the vials was observed in the samples with the NE and subjected to retrofilling, which may be due to the production of water through transesterification reactions in the cellulose. In the LM group, a similar behaviour to the NE-immersed samples was observed for the water. The mass of water in the vial gradually decreased over time for the first part of the ageing process (Figure 8a), although the decrease was not as sharp as in the HM group. In the case of the NE-impregnated samples, the most-significant drop occurred after 1000 h of ageing, compared to the HM group, where it occurred at 500 h. It is important to note that the decrease in water content in the MO samples was not as pronounced as in the HM group.

To complete the reasoning, the evolution of the acidity can be observed. As Figure 10a shows, during the initial 500 h of ageing, the acidity of the NE did not exhibit a significant rise. However, after 750 h of ageing, a sharp increase in the acid value was observed, multiplying its value by 55 in the NE-immersed samples. When comparing this trend to Figure 7b, it becomes apparent that the aforementioned sharp rise in the moisture content of the oil occurred around the same ageing time. Subsequently, after 1000 h of ageing, both the acid number and the moisture content stabilised. This observation suggests that the fatty acids resulting from hydrolysis were being consumed, which is in agreement with the previous reasoning and suggests that transesterification became the predominant reaction at that stage of the experiment. Transesterification generates water and reinforces the cellulose structure, as indicated by the steady levels of the *TS* content (Figure 4a) and the *DP* value (Figure 5a) for the NE-impregnated Kraft paper after 750 h of ageing.

A similar reasoning can be applied to the acidity of the low-moisture samples subjected to retrofilling (Figure 10b), as the parameters followed a similar trend, although the tendencies were less. As was explained, the acidity of the NE-immersed group increased, but much less than for the HM samples. This led to the conclusion that the moisture of the paper had an effect on the acidity of the oil, possibly due to the lower generation of fatty acids in the hydrolysis reaction. This increase was also noticeable in the retrofilled samples, but with the delay of having changed the oil. Another difference with the other group of samples was that, at the end of the test, there was not a clear stabilisation of the acid value of the oil.

The observation of all the parameters measured in the process suggests that the solid insulation of retrofilled transformers would follow a similar trend as that of the NE-immersed insulation. The ageing rate was lower because of the drying effect of the ester and the appearance of transesterification on the cellulose structure.

## 6. Conclusions

This paper presented a study involving the accelerated ageing of Kraft paper samples subjected to retrofilling. The samples were first impregnated with MO and subjected to accelerated ageing in an oven. Afterwards, they were extracted from the oven and retrofilled with NE. Comparable samples were also created and impregnated with MO and NE; those samples were subjected to the same ageing conditions without replacing the oil. Samples with two different moisture contents were tested to understand the influence of moisture in the ageing process.

The results indicated that, after retrofilling, Kraft paper tended to exhibit similar behaviour to NE-impregnated Kraft paper for most of the measured parameters, with a decrease in the degradation rate as confirmed by the *DP* and *TS* tests. The measurements of the water content, in the paper and oil, along with the acid value, facilitated the observation of the hydrolysis and transesterification reactions in the NE-filled and retrofilled samples. The measurements demonstrated that both reactions occurred in the retrofilled samples, although with a certain time delay compared to the NE-impregnated samples. The results suggested that the impregnation of the paper with the MO did not have a significant effect on the ageing reactions that took place in the retrofilling scenario.

The results of the study suggested that the ageing rate of the Kraft paper would be slowed down when it is subjected to retrofilling, indicating that retrofilling with NE may extend its life expectancy. However, even if the experimental study proved that the ageing process of Kraft paper retrofilled with NE slowed down, there are still doubts regarding the remaining parameters of insulation operation in power transformers, e.g., partial discharge, thermal conductivity, ageing products, etc. More research is required to investigate the real impact of the retrofilling on the life expectancy of a transformer.

## Figures and Tables

**Table 1:** Sampling times for the high-moisture (HM) and low-moisture (LM) samples (the samples extracted at each testing time are marked with “x”).

Ageing Time (h)	HM (4.5%)			LM (1%)		
	**MO**	**NE**	**RF**	**MO**	**NE**	**RF**
0	x	x		x	x	
96	x	x				
218	x	x	x	x	x	
288			x			
386			x	x	x	x
458	x	x	x			x
552			x			x
627			x	x	x	x
793	x	x	x			x
959				x	x	x
1060			x			
1226						x
1275	x	x	x			
1441				x	x	x
1567	x	x				
1733				x	x	
1802	x		x			
1968				x	x	x
2067			x			x
2217	x	x				
2383				x	x	x
Number of samples	9	8	11	9	9	12

**Table 2:** Condition of the Kraft paper before the retrofilling.

	High-Moisture Samples	Low-Moisture Samples
*TS* retained	36.1%	71.1%
*DP* retained	43.8%	71.8%

**Table 3:** Parameters obtained when fitting the *TS* results to (1).

$\mathrm{TS}=a\cdot e^{\left(b\cdot t\right)}+c\cdot e^{\left(d\cdot t\right)}$		*a*	*b*	*c*	*d*	*R* ${}^{2}$
HM	MO	1.598	−0.00829	1.586	−0.00460	0.9994
	NE	2.653	−0.0059	0.4779	−7.504 $\times \;10^{-5}$	0.9791
	Retrof	3.074	−0.00484	0.175	0.00031	0.9254
LM	MO	2.668	−0.00202	0.0453	0.00063	0.9904
	NE	2.814	−0.00141	0.0952	0.00063	0.9981
	Retrof	3.95	−0.00212	0.0732	0.00056	0.8765

**Table 4:** Parameters obtained when fitting the *DP* results to (2).

$\mathrm{DP}=a\cdot e^{\left(b\cdot t\right)}+c\cdot e^{\left(d\cdot t\right)}$		*a*	*b*	*c*	*d*	*R* ${}^{2}$
HM	MO	758.9	−0.006565	232.4	8.014 $\times \;10^{-5}$	0.9989
	NE	615	−0.00696	468	−2.389 $\times \;10^{-5}$	0.9601
	Retrof	443.4	−0.001096	86.46	0.000537	0.8302
LM	MO	623.2	−0.00265	360.4	−0.000176	0.9829
	NE	831.7	−0.00231	409.6	6.119 $\times \;10^{-5}$	0.9747
	Retrof	679.9	−0.00053	28.41	0.0008959	0.693

**Table 5:** Parameters of Pearson’s linear correlation between *DP* and *TS*.

TS (N/mm) = a·DP+b		*a*	*b*	*R* ${}^{2}$
HM	MO	0.0038	−0.5813	0.974
	NE	0.0045	−1.765	0.960
	Retrof	0.004	−0.885	0.586
LM	MO	0.0036	−0.7126	0.981
	NE	0.0031	−0.8135	0.952
	Retrof	0.0043	−1.1937	0.477

## Data Availability

The data presented in this study are available on request from the corresponding author.
